# Effect of Guar Gum with Sorbitol Coating on the Properties and Oil Absorption of French Fries

**DOI:** 10.3390/ijms18122700

**Published:** 2017-12-13

**Authors:** Bo Jia, Daming Fan, Jinwei Li, Zhenhua Duan, Liuping Fan

**Affiliations:** 1 Institute of Food Research, Hezhou University, Hezhou 542899, China; 6150111015@vip.jiangnan.edu.cn (B.J.); fanliuping@jiangnan.edu.cn (L.F.); 2 School of Food Science and Technology, Jiangnan University, Wuxi 214122, China; fandm@jiangnan.edu.cn

**Keywords:** blanching, frying, French fries, oil distribution, guar gum, sorbitol

## Abstract

This paper investigated the effects of guar gum with sorbitol coating on the oil absorption of French fries by combined dye oil methods, confocal laser scanning microscopy (CLSM) and scanning electron microscopy (SEM). The results showed that pretreatment of blanching with calcium ions and coating with guar gum and sorbitol could significantly reduce the structural oil (STO) and penetrated surface oil (PSO) of French fries and have no negative effects on its texture and also effectively control the final moisture content (*p* < 0.05). Compared with control or samples coated with guar gum (blanching with or without calcium ions), the total oil (TO) of French fries with guar gum and sorbitol reduced by 50.8%, 33.1% and 30.6%, respectively. CLSM photographs confirmed that STO significantly reduced after coating with guar gum and sorbitol, followed by PSO. In the process of frying, the coatings of guar gum or guar gum with sorbitol could effectively prevent oil from infiltrating the potato tissue, which can be seen in the SEM photographs. The barrier properties of French fries were enhanced by coating guar gum, and sorbitol was added to avoid pores and cracks. Blanching with calcium ion can significantly reduce the final moisture content of coating French fries.

## 1. Introduction

Frying is a complex process involving simultaneous heat and mass transfer, which is typically in the range of 150 to 200 °C [1]. Frying can improve the quality of food so that it has a unique sensory characteristics used in food industry and food services industry, frying is one of the traditional and popular processing methods [2]. French fries are popular with consumers because of their pleasant taste and typical flavor [3]. French fries generally produced by immersion in edible oil or fat have a relatively high oil content. Excessive intake of oil greatly increase the risk of adverse health outcomes such as obesity, high blood pressure and coronary heart disease [4], thus reducing the oil content of French fries is among the most important requirements.

There are numerous researchers who found that oil absorption was closely related to water evaporation during frying. Oil content decreased as the initial moisture content decreased [5]. Amounts of researches were related to the reduction of oil content during frying by using pre-drying [6], optimization of frying condition [7], and edible coatings [8]. Pre-drying before frying could reduce the initial moisture content of the food and reduce the oil content [6]. The oil content of potato chips was influenced by frying temperature and the type of oil used for frying [7]. The effect of oil reduction was very limited by optimization of frying condition. Pre-drying leads to high energy consumption and high cost and can also cause the deformation and chemical changes of food material [9]. Compared to the previous two methods, application of coatings is an alternative, economical and safe method to reduce the oil content of French fries.

Some edible coatings, particularly hydrophilic polymers, have the desirable barrier properties to gas/moisture and good mechanical characteristics, which gives them potential to decrease oil uptake, to prevent moisture loss, preserve texture, and maintain color, consequently extending the product shelf-life in fried products [10]. Since the surface properties of foods affect the oil absorption during frying, coatings make the surface stronger and more compact, with fewer less porous, the surface modification by the coatings can contribute to reduce water evaporation and leads to less oil uptake; also, coatings alter the water-holding capacity by trapping moisture inside and preventing the replacement of water by oil [11]. Additionally, hydrocolloids can be used as emulsifiers in composite films [12], the surface tension between the oil and the food could also be reduced, consequently contributed to decrease oil uptake. The mechanisms of the oil uptake during frying are associated with heat transfer from the frying oil to the food. The hydrocolloid coatings significantly reduced the heat transfer coefficients as well as oil uptake which became more apparent at higher concentrations [13]. Thus, reducing the oil content of French fries by application of coatings is an effective method.

Among the hydrocolloids used for coating foods for frying, the use of guar gum has been found to be effective; guar gum is a natural polysaccharide with non-toxicity, safety, biodegradability, biocompatibility, renewability, cheaper prices and availability properties [14]. Deok Nyun Kim et al. [13] found that guar gum had a good effect on reducing oil and oil content decreased by 40%. Extensive researches have demonstrated that plasticizers can improve the mechanical properties of biodegradable films. Films containing low-molecular weight plasticizers can reduce tensile strengths, which may make them suitable for applications that require better gas or vapor barrier properties [15]. Garcıa et al. [16] noted that an edible methylcellulose coating plasticized with sorbitol on potato strips and dough discs caused an oil reduction of 40.6 and 35.2%, respectively. Tavera-Quiroz et al. [17] found that the addition of sorbitol enhanced the barrier properties of an edible methylcellulose, and reduced the oil uptake by 30% with respect to uncoated samples during the deep fat-frying. By combining hydrogen bonds with polymers and reducing polymer interactions, the coatings have better adhesion and flexibility, and reduce the possibility of discontinuity and brittleness [18,19]. Compatibility between plasticizer and polymer is necessary for effective plasticization, which could be featured by polarity, hydrogen bonding, dielectric constant, and solubility parameters [20]. Polysaccharide-based films are commonly plasticized with polyols such as sorbitol. The plasticizer of sorbitol could improve the films flexibility and lower tensile strength and higher elongation at break [17].

Khalil [21] reported that potato strips coated with a combination of 0.5% calcium chloride and 5% pectin had the highest reduction of oil content. Simundic et al. [22] found that 0.5% of the calcium chloride blanching with the carboxymethyl cellulose (CMC) film of 1%, with the best effect of reducing oil. However, it was found that the final moisture content was also increased. For instance, Durán [23] and Moreno et al. [24] both claimed that soaked with 20% sodium chloride, so that the material was dehydrated to reduce the moisture content of the material before frying, in order to control the oil content. Bunger et al. [25] reported that soaking with 3% NaCl solution for 50 min significantly reduced oil uptake. But, it need to know that the decrease in oil absorption using osmotic dehydration pretreatment was attributed to the increase in solids content occurring during the osmotic dehydration process rather than a reduction in the amount of oil taken up [24].

However, researchers were more concerned with the reduction of the total oil content of coated samples. Whether the coating affected the oil distribution or oil fraction of French fries was not clear. Furtherly, there are few reports focused on the effect of coating on the final moisture content of fried samples. As a consequence, one aim of the present study was to determine the effect of guar gum with or without sorbitol on the quality, oil fraction, oil reduction, and texture of French fries and study the effect of blanching with calcium ion on the final moisture content of French fries coating with guar gum. Furthermore, the effect of coatings on the oil distribution patterns and the microstructure of French fries was also investigated during frying by the dye oil method, confocal laser scanning microscopy (CLSM), and scanning electron microscopy (SEM).

## 2. Results and Discussion

### 2.1. Initial Water Content, Solid Content, and Starch Content of Potato

The changes of the initial water content, solid content and starch content of potato slice after blanching was shown in Table 1. Compared with the raw potato slice, the water content of potato samples after blanching increased from 81.5 g/100 g (wb) to 83.7 g/100 g (wb), the solid content of potato samples decreased from 18.5 g/100 g (wb) to 16.3 g/100 g (wb). However, blanching treatment have no significant effects on the initial water content and solid content of potato slice (*p* > 0.05). The initial starch content of the potato samples significantly decreased from 13.1 g/100 g (wb) to 10.9 g/100 g (wb) after blanching. It turned out to be 10.9 g/100 g (wb) after blanching (Table 1), and the starch content reduction was 16.7% (*p* < 0.05).

### 2.2. Effect of Coating on the Water Content and Oil Content

As shown in Table 2, compared with the control, coating with guar gum treatments significantly improved the water content and reduced the oil content of French fries (*p* < 0.05). Blanching add calcium ion and applying guar gum, guar gum and sorbitol on French fries produced a reduction of TO absorption against the non-fat total solid content by 29.1% and 50.8%, respectively, compared with control treatment. When the TO of French fries expressed against the non-fat potato solid content, blanching add calcium ion and applying guar gum, guar gum and sorbitol on French fries produced a reduction of oil absorption by 20.7% and 35.7%, respectively, compared with control treatment. It indicated that plasticized guar gum with or without sorbitol on French fries could really reduce the TO, not just owing to the increase in solid content. Garmakhany et al. [26] found that potato chips coating with 0.3% guar gum could reduce the oil content but increase the moisture content. The food products coating the guar gum has the ability to affect the moisture migration. This is consistent with our results, but as we can see, when the French fries blanching with calcium ion then coating with guar gum could effectively reduce the final moisture content of fried samples and contribute to the reduction of oil content.

Kim et al. [13] reported that coating French fried potatoes with different concentrations of 0.3%, 0.6%, 0.9%, and the effect of 0.9% guar gum was significant and reduced oil uptake by 40%. Sothornvit [27] found that coating banana chips with guar gum, producing a reduction of oil absorption by 25.2% compared with control samples. Yu et al. [28] found that compared with control potato chips with 1% guar gum and glycerol produced a reduction of oil absorption by 51.8%. The oil contents in these researches all expressed against the total solid content, which was related to the oil absorption during frying. Based on this, the following oil content was all expressed against the non-fat total solid content, which was independent to the oil absorption.

Compared to guar gum coated sample without plasticizer and blanched without calcium ion, calcium ion addition in blanching showed noticeable differences with regard to water content (*p* > 0.05), and sorbitol addition made the TO significantly reduce, turned out to be 50.8% (Table 2). On the one hand, the addition of calcium ions can make the water evaporation better during frying, on the other hand, the addition of sorbitol can effectively prevent the absorption of oil of fried samples. Compared to guar gum coated sample without plasticizer but blanched with calcium ion, sorbitol addition did not show noticeable differences with regard to water content, but the TO significantly reduced by 30.6%. The addition of sorbitol as a plasticizer at the correct level might could reduce intermolecular forces and increase the mobility of polymer chains, which could improve the mechanical properties of edible films [17].

Oil reduction of the fried samples could be attributed, among other causes, to the differences in adhesion with substrate, surface characteristics of the sample, frying conditions, and different frying processes [26,29]. Low quantities of guar gum in aqueous solution can confer high viscosity, and even 1% aqueous dispersion of good quality guar gum may possess viscosity as high as 10,000 cP [30]. The good adherence of the guar gum coating to the surface of French fries might have resulted from the high viscosity. On the other hand, Vina et al. [31] observed that the addition of a plasticizer decreased the surface tension, facilitating coating adhesion to foodstuffs. According to Tavera-Quiroz et al. [17], the application of coating plasticized with sorbitol was an effective choice to reduce oil absorption in fried potato, the presence of sorbitol improved the flexibility and mechanical properties of the films and their integrity. Albert and Mittal [32] reported that separate coating had some holes after frying and the addition of a plasticizer was necessary. As the founding of Patsioura et al. [33], the shrinkage or damage of cell walls during slicing and superheated drying created larger passages to oil. The coating treatment could create a layer of film, which hinder the oil from entering into the passages created by frying. In the other hand, blanching with calcium ion then coating guar gum with or without sorbitol, which decreases the incorporation of oil in French fries and helps to evaporate the water during the frying operation.

### 2.3. Effect of Coating on Oil Fractions of French Fries

**Figure 1:**
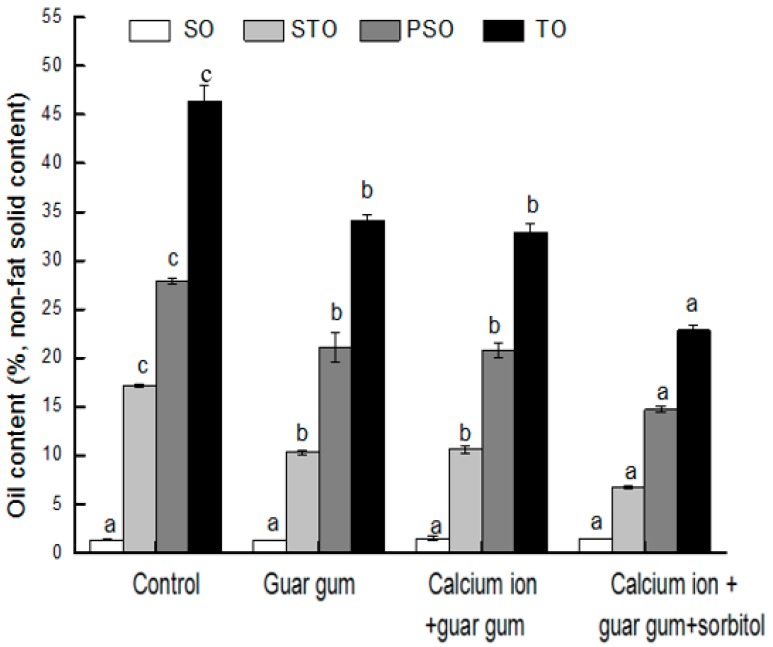
Effect of coating treatments on total oil content and oil fractions of the French fries. Data values are means ± SD (*n* = 3, *N* = 12). The same letter in column means no significantly different (*p* > 0.05).

Oil fractions and TO in the fried product depended on the different coating treatments as shown in Figure 1. The SO content of final French fries did not show noticeable reduction with different coating treatments (*p* > 0.05), but TO, PSO and STO of French fries diminished significantly (*p* < 0.05). Compared with control sample, the PSO content of three kinds of coated French fries reduced 24.14%, 25.45% and 47.28%, respectively, and the STO content by 39.97%, 38.23% and 61.24%, respectively. Thus, the TO reduction of French fries was mainly due to the reduction of STO. During frying, the initial frying stage is the main stage that affects oil absorption. The coating strengthened the surface of the sample to effectively prevent the absorption of oil. After the chips are removed from the fryer, the higher temperature difference between the surface and the interior generated the higher negative pressure in the pore space, resulting in more oil penetration into their microstructures [34,35]. The film acted as a barrier to hinder oil penetration, which may lead to the reduction of PSO. Consequently, coatings enhance the barrier properties of French fries during frying and cooling, and avoid pores and cracks in the fried products.

**Figure 2:**
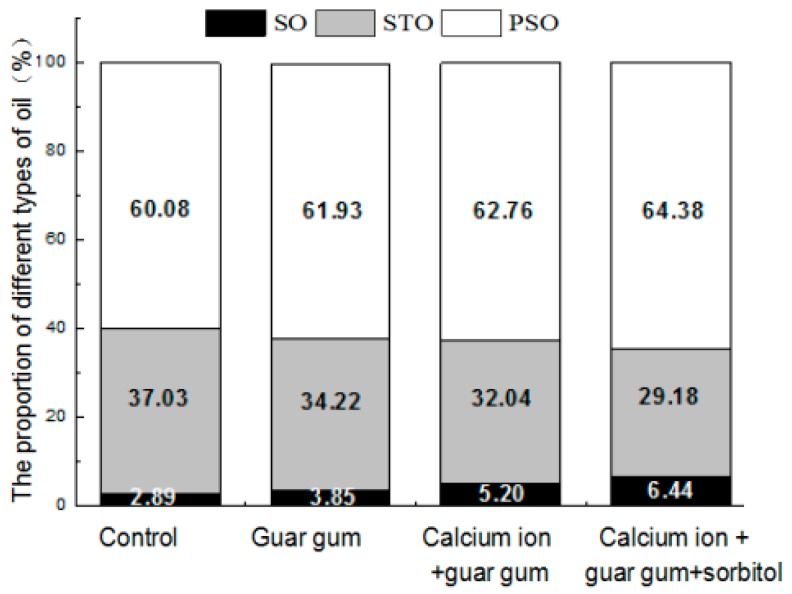
Effect of blanching and coating treatments on the proportion of different types of oil of the French fries.

PSO constituted the highest fraction of TO during frying of both control and coated French fries. Coating treatment not only reduced the oil content, but also varied the oil percentage in TO. The percentages of PSO based on the TO content were 60.08%, 61.93%, 62.75% and 64.39% for control and three kinds of different coating treatments, respectively (Figure 2). This fact suggests that oil absorption in French fries is mainly a surface phenomenon, which is related to the equilibrium between the adhesion and drainage of oil [34]. The STO fraction is the second important fraction in the TO content during frying of the control as well as the coated potato strips. The percentage of STO based on the TO content were 37.03%, 34.22%, 32.04%, and 29.18% for control and three kinds of different coating treatments, respectively. This confirms that a little of oil penetrates into fried products. SO was the lowest constituent of TO content, the percentage of SO based on the TO content were 2.89%, 3.85%, 5.20%, and 6.44% for control and three kinds of different coating treatments, respectively. In general, there was no significant difference in SO content (*p* > 0.05).

### 2.4. Oil Distribution and Microstructure of French Fries

Compared with the classical microscopy, CLSM could observe the optical sections at different depths of the samples and investigate oil distribution in the crust as close to the real situation as possible [36,37].

**Figure 3:**
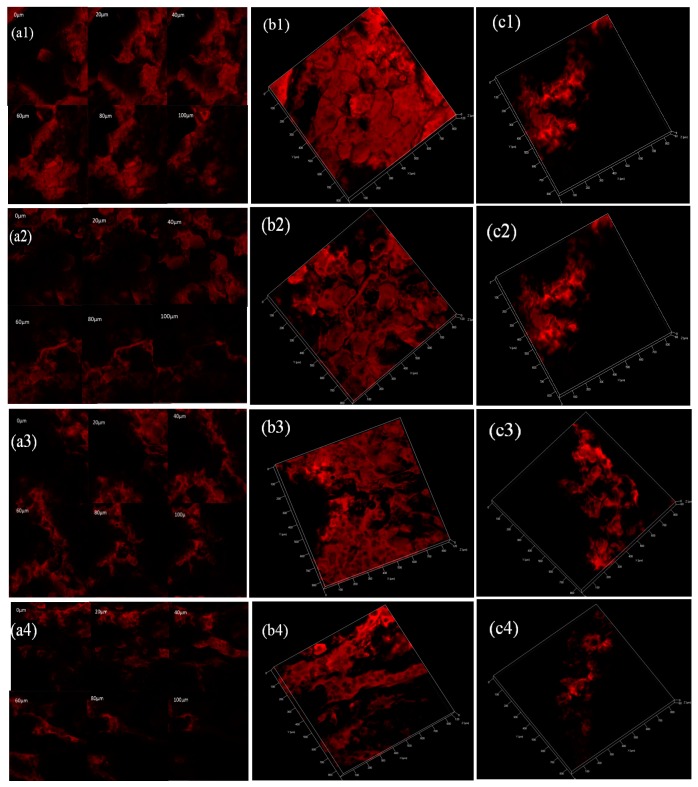
Fluorescence mode confocal laser scanning microscopy (CLSM) images of oil distribution in control (1), guar gum coated (2), blanching with calcium ion and guar gum coated (3), plasticized guar gum coating with sorbitol (4) of French fries. (**a**) Gallery of CLSM images at different depths; (**b**,**c**) 3D reconstructions of CLSM galleries; (**b**), the surface of contact with oil; (**c**), opposite to the surface of contact with oil. Each image in the gallery and the 3D image: 1024 × 1024 μm.

Figure 3 (1), (2), (3), and (4) show different patterns of oil distribution in control and coated samples, respectively. Figure 3(a1–a4) show that images were taken every 20 μm as the laser penetrated down the *z* axis in French fries. Only the oil which is in focus at different depths in the French fries is recorded in the image. The 3D reconstructions of the gallery of images were obtained in Figure 3(b1–b4). As shown in Figure 3, the oil mainly covered the intercellular spaces and cannot enter the integrated cell interior, which is consistent with the results of Bouchon and Aguilera, Pedreschi et al. [37,38]. They observed that the oil seemed to flow through the intercellular space and does not entered the cell interior. The red area stands for the oil distribution of French fries and the larger the red area is, the more the oil content is. The red area of the control was the largest and it nearly occupied the whole French fries (Figure 3(b1)). Compared with control, coated with guar gum effectively reduced the red region of French fries and some free oil region could be found (Figure 3(b2,b3)). Blanching treatment with or without the calcium ion have no significant effect on the oil content of French fries coating with guar gum (*p* > 0.05). The French fries coated with guar gum and sorbitol had the largest free oil region and more uniformly distribution of oil (From Figure 3(b4)).

As shown in Figure 3(a1), the red region near surface of sample was limited and it enlarged with the depth increasing, which is consistent with the result of the oil fraction in fried French fries, PSO constituted the highest fraction of TO during frying. Figure 3(a2–a4) show that the oil is not uniformly located at each depth, thus, oil appeared to be not concentrated in the entire region. Compared with control sample, the interior layers of French fries coating with guar gum are free of oil (Figure 3(a2,a3)), this result confirms that at least some of the oil does not remain in the outer layer of a French fries, the guar gum coating have the ability to be good lipid barrier and decrease the STO content and oil uptake in fried French fries. Compared to guar gum coated sample, sorbitol addition shows noticeable differences with regard to the oil location (Figure 3(a4)), suggesting that some of the oil does not penetrate deeply into the interior of the French fries coating with guar gum and sorbitol and reduce the STO and PSO content. Sorbitol addition improved the flexibility and handling of guar gum coating, maintained integrity, and avoided pores and cracks in the fried French fries.

As shown in Figure 3(c1–c4), show oil distribution of the surface opposite to the surface of contact with frying oil in control and coated samples, respectively. The larger the red (in the web version) area is, the more the oil content is. Most of the oil distribution was found over the entire region (Figure 3(c1)). Compared with control sample, the red area in French fries coated with guar gum diminished with some free oil region (Figure 3(c2,c3)). Compared to the guar gum coated sample, sorbitol addition shows noticeable differences with regard to the oil distribution (Figure 3(c4)). It turned out that the use of guar gum could significantly hinder the penetration of oil during frying [13,26,27], and sorbitol addition could improve the effect of reducing oil.

**Figure 4:**
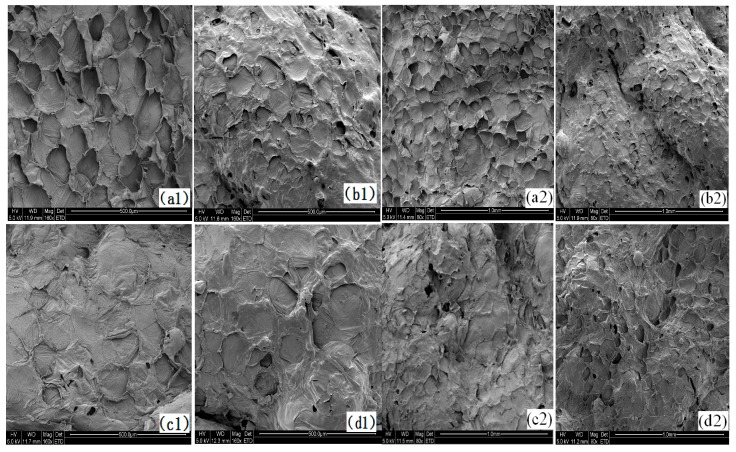
Scan electron microscope photograph of French fries (**a1**,**a2**) means control; (**b1**,**b2**) means coating with guar gum; (**c1**,**c2**) means blanching with calcium ion coating with guar gum; (**d1**,**d2**) means blanching with calcium ion coating with guar gum and sorbitol; **left** means ×160, **right** ×80.

The SEM photographs of French fries (Figure 4), control, coated with guar gum, and coated with guar gum and sorbitol were compared. It might explain the results of oil distribution and content. The control product did not show extensive cell separation and ruptured cells. In contrast, maintained the integrity of most cells. This was different with the results reported by Khalil, Singthong, and Thongkaew [11,21].

The photographs of French fries coated with guar gum and coated with guar gum and sorbitol look smoother than those of control samples (Figure 4), consequently preventing oil penetration into the potato tissue during frying process. Compared to samples coating with guar gum, blanched without calcium ion samples showed reduction in cell volume and modified the shape as well (Figure 4b,c). This may be due to the sample without blanched with ion is not conducive to the escape of moisture, resulting in a strong vapor pressure, which leads to cell deformation. As we can see from the results of oil fraction, SEM and CLSM, the total solid content and the surface structure has a decisive effect on the oil absorption of sample. The treatment of coating with guar gum could decrease the oil content of French fries, but it could also partly prevent the water evaporation of samples during frying, which was related to the shrinkage of the sample surface (Figure 4b).

### 2.5. Texture of French Fries

Table 3 showed the hardness of French fries produced by different methods. Hardness is an important index for the French fries because there is a certain relationship between the hardness and chewiness of French fries. The hardness and chewiness were different for French fries produced by different methods, but the differences were not significant, suggesting different treatments had no significant effect on their texture. Meanwhile, it was also observed that the sample hardness decrease coating with guar gum. The sample coating with guar gum might hinder internal moisture vaporation; thus, the sample became soft so that it is hardness and chewness decreased. Among other treatment, the internal moisture was evaporated rapidly, which created a low final moisture content that increased the sample hardness.

The lightness parameter L* is commonly used as a quality control parameter for fried foods [39]. Higher L* value indicates a lighter brightness, which is favorable for fried foods. Table 3 showed the color values of French fries produced by different methods. It was reported that the L* values were decreased with frying time increasing [25,40], and the longer the frying time, the more the non-enzymatic browning reactions occur. Under different conditions, the L* value increased, but there was no significant difference. Table 3 also showed that the control group possessed the highest yellowness (b*) value, suggesting they had stronger golden yellow color. This could also be related to the frying process. The control group would lead to more oil penetrated into the sample and more chemical oxidation, and eventually led to stronger golden yellow color. Overall, it means that coating does not influence the texture of French fries. It is possible to reduce oil content of this kind of potato product with no consequences on its quality, by coating with guar gum with or without sorbitol.

**Figure 5:**
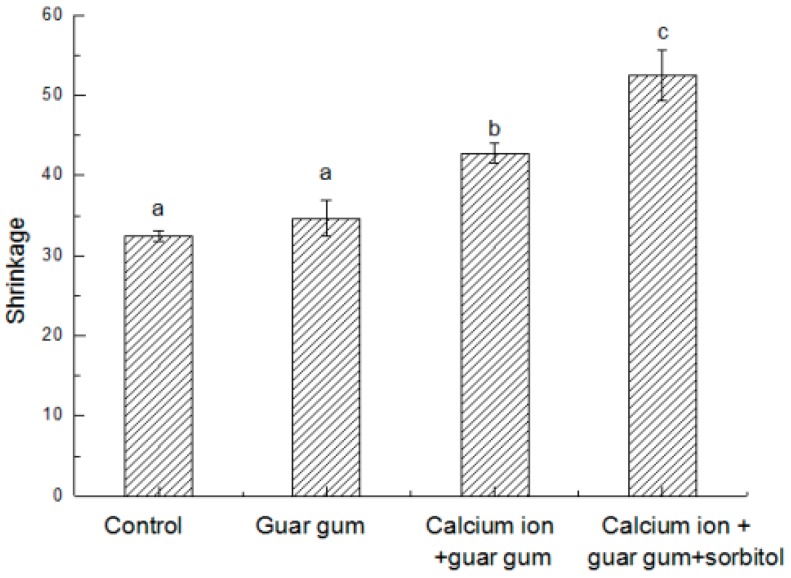
Comparison on the shrinkage of French fries produced by different methods.

Maadyrad et al. [41] reported that volume shrinkage during early stages of frying was nearly equal to the volume of water loss. Therefore, the volume of shrinkage is depend on the evaporation of the water in the product and the amount of oil absorbed. As shown in Figure 5, the volume shrinkage took place in all French fries due to water loss, but the shrinkage degree of control group was smaller, which may be resulted from there is no film lead to more evaporation of water and oil infiltration, thus producing a resistance to volume change. Compared with control treatment, coating with guar gum can prevent the evaporation of water, but also effectively prevent the infiltration of oil, resulting in increased shrinkage, but there was no significant difference (*p* > 0.05). When blanching with calcium ion and guar gum coating with or without sorbitol, the final volume shrinkage significantly increased by 31.9% and 54.7%. This was mainly due to increased moisture evaporation. Meanwhile, there was a small amount of oil absorption. The generated interior force was too low to make enough internal moisture evaporation and vapor pressure.

## 3. Materials and Methods

### 3.1. Materials

Potatoes (*Solanum tuberosum* L.) and palm oil (produced by Jia-li Co., Ltd., Shanghai, China) were purchased from a supermarket in Wuxi, China. The potato samples were stored in a refrigerator at 4 °C until they were used. Sudan I (Sinophrm Chemical Reagent Co., Ltd., Shanghai, China) was used to dye oil to distinguish different oil fractions. Nile Red (Tokyo Chemical Industry Co., Ltd., Tokyo, Japan) was a fluorescent agent used to stain oil for observation of CLSM.

### 3.2. Sample Pre-Treatment

The potato tubers were sorted, washed, peeled, and cut into slices (8 mm × 8 mm × 60 mm), these slices were blanched in deionized water with or without calcium ions for 4 min at 95 °C, then cooled to ambient temperature with a flow of cold water. The excess water was blotted out using lint-free tissue paper.

The guar gum (Henan Baikang Chemical Co., Ltd., Zhengzhou, China) concentration of 1% (*w*/*w*), and the sorbitol (Sinopharm Chemical Reagent Co., Ltd., Shanghai, China) concentration of 3% (*w*/*w*) were prepared. 45 g potato strips were immersed into 300 mL guar gum solution with or without sorbitol at 40 °C. After 1 min immersion, the sample was drained on a wire structure to remove the redundant coating solution for about 20 min. Each experiment was tested in triplicate.

### 3.3. Frying Procedure

Oil dyed with Sudan red preparation: the 1 g Sudan I is completely soluble in the 1 L Palm oil, then the oil was diluted into different concentrations using petroleum ethe (PE) (Sinopharm Chemical Reagent Co., Ltd., Shanghai, China). Their absorbance was measured at the maximum absorption wavelength of 459 nm using a spectrophotometer (UV2600, Shanghai Tian Mei Scientific Instruments Co., Ltd., Shanghai, China) at room temperature.

Frying procedure: Different drying and control samples were fried in a thermostatically temperature controlled fryer (Jintan Precision Instruments Co., Ltd., Changzhou, China) filled with 3 L of Palm oil. The potato weight/oil volume ratio was 1:30. The frying temperature was 170 °C for 6 min. Twenty seconds before ending each frying process, 0.188 L of dyed Sudan Red I was added quickly [28].

## 4. Analytical Methods

### 4.1. Water Content, Solid Content and Starch Content

The water content was measured in a convective oven at 105 °C until constant weight was arrived. Each sample was tested at least in triplicate. Starch content of the potato was determined according to the National Standard GB/T 5514-2008 in China. For each coating, each sample was tested at least in triplicate.

Solid content was calculated as follows:

Solid content (% wb) = 100% − Initial water content (% wb)(1)

### 4.2. Determination of Oil Content

Oil content and oil fraction of French fries were analyzed by the method of Yu et al. [28] and Zhang et al. [42]. The total oil (TO, %) of French fries was determined by the Soxhlet method and expressed against the non-fat solid content.

The oil fraction of French fries were analyzed by dye oil (Sudan Red I, Sigma Chemicals, St. Louis, MO, USA) methods. The surface oil (SO, %) content of French fries was determined by immersing French fries in petroleum ether for 1 s at ambient temperature and dried to constant mass. The penetrated surface oil (PSO, %) content and the structural oil content (STO, %) of French fries were calculated as follows:

(2) $$PSO\left(\%\right)=\frac{\mathrm{dye}\;\mathrm{oil}\left(g\right)\times \mathrm{dye}\;\mathrm{concentration}\;\mathrm{in}\;\mathrm{extracted}\;\mathrm{oil}\left(g/L\right)}{\mathrm{dye}\;\mathrm{concentration}\;\mathrm{in}\;\mathrm{oil}\;\mathrm{bath}\;\left(g/L\right)\times \;\mathrm{non-fat}\;\mathrm{solid}\left(g\right)}\times 100\%$$

(3) STO(%)=TO(%)−SO(%)−PSO(%)

### 4.3. Determination of Color

The color parameters (L*, a*, b*) of the samples were measured using a CR-400 Chroma Meter (Konica Minolta Sensing Inc., Tokyo, Japan), which had been calibrated with a standard whiteboard. Parameters L*, a*, and b* indicate the intensity of lightness, redness, and yellowness of the sample, respectively. Each sample was measured three times.

### 4.4. Determination of Texture

The texture was determined using a texture analyzer (TA-XT plus, Stable Micro Systems, Ltd., Surrey, UK) fitted with a stainless-steel ball probe (P/0.25 s). The pre-speed, test-speed, and post-speed were set as 1.0, 5.0, and 5.0 mm/s, respectively.

### 4.5. Microscopic Analysis

The inverted CLSM (Carl Zeiss, Oberko, Germany) was used to observe the oil distribution and the surface morphology of the French fires. Nile red (Tokyo Chemical Industry Co., Ltd., Tokyo, Japan) was mixed with the hot oil at a concentration of 0.0192 mg/mL during frying [28,42]. The serial image of French fries was integrated to 3D images by Carl Zeiss LSM software (3.92, Oberko, Germany).

SEM was used to examine the surface of French fries. Coated and uncoated potato samples were examined on a Hitachi S4800 SEM (Tokyo, Japan) at an accelerating voltage of 5.0 kV [28].

### 4.6. Statistical Analysis

All these experiments were carried out in triplicate. Data was analyzed using SPSS software version 17.0 for Windows (SPSS 17.0, IBM, Chicago, IL, USA). Difference among the mean values of French fries was determined using one-way analysis of variance, and the least square difference test (LSD) is significant different at *p* < 0.05.

## 5. Conclusions

Coating with guar gum and sorbitol could effectively hinder the oil absorption of French fries and have no negative effects on its texture. Compared with control or samples coated with guar gum (blanching with or without calcium ions), French fries with calcium blanching and guar gum + sorbitol coating produced a reduction of oil absorption by 50.8%, 33.1%, and 30.6%, respectively. Both for control or coated French fries, penetrated surface oil (PSO) was dominant in total oil (TO), followed by structural oil (STO) and surface oil (SO). Coating treatment with guar gum and sorbitol and blanching with calcium ions not only can significantly reduce the STO and PSO of French fries but also effectively control the final moisture content (*p* < 0.05). STO was the main factor attributed to the TO reduction of French fries after coating. Oil absorption in French fries is, to a large extent, dependent on the microstructure and surface properties of samples. Coating formulations enhance the barrier properties of French fries and avoid pores and cracks in the fried products with higher toughness. 

CLSM photographs confirmed that some of the oil was trapped in the outer layer of the French fries coating with guar gum, and some of the oil does not penetrate deeply into the interior of the French fries coating with guar gum and sorbitol. SEM photographs indicated that guar gum and guar gum and sorbitol coatings were effective in preventing oil penetration into the potato tissue during frying process.

The main components of the coating formulation are water, guar gum and sorbitol, which are safe and inexpensive and could be conveniently used during the production of French fries.

## Figures and Tables

**Table 1:** Initial water content, solid content and starch content of potato before and after blanching *.

Material	Initial Water Content (% wb)	Solid Content (% wb)	Starch Content (% wb)
Raw potato	81.5 ± 0.4 ^a^	18.5 ± 0.4 ^a^	13.1 ± 0.2 ^a^
Bleached potato	83.7 ± 3.7 ^a^	16.3 ± 3.7 ^a^	10.9 ± 0.1 ^b^

* Data values are means ± SD (*n* = 3, *N* = 6). The same letter (^a^ or ^b^) in column means no significantly different (*p* > 0.05).

**Table 2:** Effects of coating treatments on water content and total oil content *.

Coating Treatments	Water Content (% wb)	Total Oil Content (against the Non-Fat Total Solid Content, %)	Total Oil Content (against the Non-Fat Potato Solid Content, %)
Control	59.66 ± 0.47 ^a^	46.41 ± 1.69 ^a^	46.41 ± 1.69 ^a^
Guar gum	61.75 ± 0.87 ^b^	34.15 ± 0.53 ^b^	43.97 ± 0.59 ^b^
Calcium ion +guar gum	57.00 ± 1.07 ^c^	32.91 ± 0.88 ^b^	36.79 ± 1.80 ^c^
Calcium ion + guar gum + sorbitol	56.38 ± 1.34 ^c^	22.84 ± 0.25 ^c^	29.86 ± 1.42 ^d^

* Data values are means ± SD (*n* = 3, *N* = 12). The same letter (^a^, ^b^, ^c^ or ^d^) in column means no significantly different (*p* > 0.05)

**Table 3:** Effect of coating on texture and color of French fries.

Coating Treatments	Hardness	Chewiness	L*	a*	b*
Control	1902 ± 81.00 ^a^	86.09 ± 2.99 ^a^	53.35 ± 0.63 ^a^	−1.24 ± 0.02 ^a^	15.82 ± 0.68 ^a^
Guar gum	1869 ± 56.22 ^a^	85.45 ± 3.90 ^a^	53.68 ± 0.25 ^a^	−1.27 ± 0.03 ^a^	15.72 ± 0.35 ^a^
Calcium ion + guar gum	1953 ± 13.39 ^a^	89.99 ± 4.34 ^a^	53.98 ± 0.19 ^a^	−1.24 ± 0.07 ^a^	15.34 ± 0.21 ^a^
Calcium ion + guar gum + sorbitol	1964 ± 35.92 ^a^	91.03 ± 3.28 ^a^	54.36 ± 0.82 ^a^	−1.25 ± 0.06 ^a^	15.06 ± 0.05 ^a^

* Data values are means ± SD (*n* = 3, *N* = 12). The same letter “^a^” in column means no significantly different (*p* > 0.05). Parameters L*, a* and b* indicate the intensity of lightness, redness and yellowness of the sample.
